# Meta‐analysis reveals enhanced growth of marine harmful algae from temperate regions with warming and elevated CO_2_ levels

**DOI:** 10.1111/gcb.14678

**Published:** 2019-06-17

**Authors:** Karen M. Brandenburg, Mandy Velthuis, Dedmer B. Van de Waal

**Affiliations:** ^1^ Department of Aquatic Ecology Netherlands Institute of Ecology (NIOO‐KNAW) Wageningen The Netherlands; ^2^ Department of Ecosystem Research Leibniz‐Institute of Freshwater Ecology and Inland Fisheries (IGB) Berlin Germany; ^3^ Wageningen Environmental Research Wageningen University and Research Wageningen The Netherlands

**Keywords:** climate change, global warming, harmful algal blooms, ocean acidification, sea surface temperature

## Abstract

Elevated *p*CO_2_ and warming may promote algal growth and toxin production, and thereby possibly support the proliferation and toxicity of harmful algal blooms (HABs). Here, we tested whether empirical data support this hypothesis using a meta‐analytic approach and investigated the responses of growth rate and toxin content or toxicity of numerous marine and estuarine HAB species to elevated *p*CO_2_ and warming. Most of the available data on HAB responses towards the two tested climate change variables concern dinoflagellates, as many members of this phytoplankton group are known to cause HAB outbreaks. Toxin content and toxicity did not reveal a consistent response towards both tested climate change variables, while growth rate increased consistently with elevated *p*CO_2_. Warming also led to higher growth rates, but only for species isolated at higher latitudes. The observed gradient in temperature growth responses shows the potential for enhanced development of HABs at higher latitudes. Increases in growth rates with more CO_2_ may present an additional competitive advantage for HAB species, particularly as CO_2_ was not shown to enhance growth rate of other non‐HAB phytoplankton species. However, this may also be related to the difference in representation of dinoflagellate and diatom species in the respective HAB and non‐HAB phytoplankton groups. Since the proliferation of HAB species may strongly depend on their growth rates, our results warn for a greater potential of dinoflagellate HAB development in future coastal waters, particularly in temperate regions.

## Graphical abstract

Elevated *p*CO_2_ and warming may promote algal growth and toxin production, and thereby possibly support the proliferation and toxicity of HABs. Using a meta‐analytic approach we found that elevated *p*CO_2_ increased growth rates of dinoflagellate HAB species, while this was not the case for non‐HAB phytoplankton species. Warming also led to higher growth rates, but mainly for species isolated at higher latitudes. These results warn for a greater potential of dinoflagellate HAB development in future coastal waters, particularly in temperate regions.

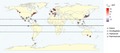

## 1 INTRODUCTION

The atmospheric partial pressure of CO_2_ (*p*CO_2_) has been rising rapidly since the industrial revolution. Due to anthropogenic activities, *p*CO_2_ has already increased from roughly 280 µatm in pre‐industrial times to ~400 µatm today (IPCC, 2014). If greenhouse gas emissions are not reduced, they are expected to cause an increase in global mean temperatures of 3–5°C by the end of this century (RCP scenario 8.5; IPCC, 2014). These changes in the Earth's climate will also impact the oceans, leading to ocean acidification and warming of the surface layers, which, in turn, greatly affect marine life (Doney, Fabry, Feely, & Kleypas, 2009; Fabry, Seibel, Feely, & Orr, 2008; Poloczanska et al., 2013). Specifically organisms living in the surface layer of the oceans, such as phytoplankton, already experience the consequences of climate change (Gobler et al., 2017; Hare et al., 2007).

Phytoplankton can become a nuisance for the environment through the formation of harmful algal blooms (HABs) that adversely affect ecosystems, fisheries, tourism and human health (Anderson, Glibert, & Burkholder, 2002; Hallegraeff, 1993). Some HAB species produce potent toxins that can accumulate in the food chain, leading to death of fish, seabirds and marine mammals. These toxins may additionally accumulate in seafood and can cause severe shellfish poisoning syndromes in humans (Wang, 2008). Non‐toxic algal blooms may also be detrimental for the environment, since they can be responsible for major reductions in oxygen concentrations in sheltered coastal areas (Zingone & Oksfeldt Enevoldsen, 2000). The development and persistence of HABs, particularly high biomass blooms, are generally expected to be promoted by eutrophication of coastal zones (Heisler et al., 2008). These blooms may possibly be further aggravated by climatic changes (Dale, Edwards, & Reid, 2006; Edwards et al., 2006; Hayes et al., 2001; Trainer et al., 2003). For instance, an increase in global temperature may enhance algal growth rates, including those of HAB species, and lead to a geographic redistribution of HABs (Anderson, Cembella, & Hallegraeff, 2012; Baumann & Doherty, 2013; Hallegraeff, 2010). Especially at higher latitudes, where the growth optima of marine phytoplankton lie well above the annual mean temperatures, warming can be expected to cause an increase in phytoplankton growth (Thomas, Kremer, Klausmeier, & Litchman, 2012). In addition, climate change may generate longer lasting seasonal temperature windows for blooms to occur (Hallegraeff, 2010). Several studies have used model simulations to link the occurrence of HABs to global temperatures, and predicted HABs to become more prominent in response to warming (Glibert et al., 2014; Kibler, Tester, Kunkel, Moore, & Litaker, 2015; Moore, Johnstone, Banas, & Salathe, 2015). Higher sea surface temperatures (SST) were also shown to be one of the facilitating factors for an increase in HAB events in both the North Atlantic and North Pacific Oceans (Gobler et al., 2017). However, changes in the occurrence and magnitude of HABs could not be explained solely by temperature and depend on additional physical, chemical and biological factors as well (Brandenburg et al., 2017; Smayda, 1997). Moreover, in regions where HAB species already grow at their temperature optimum, warming may lead to decreases in HAB events (Kibler et al., 2015).

An increase in atmospheric *p*CO_2_ may potentially stimulate phytoplankton growth indirectly, as it act as greenhouse gas and thus contributes to warming, but also directly as CO_2_ is required for photosynthesis (Beardall, Stojkovic, & Larsen, 2009). Species vary in their inorganic carbon acquisition due to differences in the operation of their carbon concentrating mechanisms (CCMs; Giordano, Beardall, & Raven, 2005). These CCMs are responsible for increasing the concentration of CO_2_ in the vicinity of ribulose‐1,5‐bisphosphate carboxylase/oxygenase (RubisCO) to ensure effective carboxylation (Badger et al., 1998; Thoms, Pahlow, & Wolf‐Gladrow, 2001). Species that rely mostly on diffusive CO_2_ uptake alone are likely to show an increase in photosynthesis and growth in response to elevated CO_2_, as they are presumably carbon limited under current *p*CO_2_ (Beardall et al., 2009; Raven, Ball, Beardall, Giordano, & Maberly, 2005). Species that utilize HCO_3_^‐ ^or rely on active CO_2_ uptake can nonetheless benefit from higher CO_2_ concentrations by downregulating their CCMs and reallocating energy and resources (Eberlein et al., 2016; Rost, Zondervan, & Wolf‐Gladrow, 2008; Van de Waal et al., 2019). Whether HAB species are to become more prominent as a direct consequence of elevated *p*CO_2_ will thus depend on their mode of carbon acquisition and the ability to regulate their CCMs.

Risks attributed to HABs depend on the magnitude and duration of blooms, which is governed by growth‐controlling factors as well as defensive traits that reduce mortality. Moreover, human health risks are strongly determined by bloom toxicity, which in turn depends on toxin production by the bloom‐forming species (Anderson et al., 2012). HAB species produce different types of toxic compounds, with distinct synthesis pathways, that may not respond similarly to climate change (Fu, Tatters, & Hutchins, 2012). For instance, domoic acid (DA) production in *Pseudo‐nitzschia* spp. has been shown to increase significantly under high CO_2_ conditions (Sun et al., 2011; Tatters, Fu, & Hutchins, 2012), whereas paralytic shellfish poisoning (PSP) toxin content has been shown to decrease in *Alexandrium catenella* (formerly *Alexandrium tamarense*; Litaker et al., 2018) in response to elevated *p*CO_2_ (Van de Waal, Eberlein, John, Wohlrab, & Rost, 2014). Yet, PSP toxin contents in another *Alexandrium catenella* strain increased with elevated *p*CO_2_ (Tatters, Flewelling, Fu, Granholm, & Hutchins, 2013), while responses reported for *Alexandrium ostenfeldii* were variable and strain dependent (Kremp et al., 2012). Furthermore, the toxicity of *Protoceratium reticulatum* attributed to yessotoxins has been shown to increase with higher temperatures (Guerrini et al., 2007; Paz, Vázquez, Riobó, & Franco, 2006), whereas *Karenia brevis* has demonstrated a higher toxicity at lower temperatures (Lamberto, Bourdelais, Jacocks, Tomas, & Baden, 2004). Thus far, it remains unclear if there is a consistent response in toxin quota and toxicity of HABs under warming and elevated *p*CO_2_, and how this may depend on the type of toxin.

A large number of studies have tested the responses of single HAB species to elevated *p*CO_2_ and warming. Here, we combined these studies using a meta‐analysis to assess the consistency of responses, and test whether marine and estuarine HAB species will grow faster and become more toxic in response to climate change. Phytoplankton species that are classified as potential HAB formers belong to different taxonomic groups. Between and within these different groups are species that can strongly differ in their physiological properties and ecological characteristics. We, therefore, also analysed climate change responses within the different phytoplankton groups. Moreover, for the CO_2_ responses, we performed this analysis for HAB as well as non‐HAB species in order to test whether HAB species might be selectively favoured by elevated *p*CO_2_. Lastly, we assessed whether the response of HAB species to warming may change along a latitudinal gradient.

## 2 MATERIALS AND METHODS

We compiled a database containing data on growth rates and toxin content or toxicity of numerous harmful algal species under various temperature and CO_2_ conditions. A literature review was first performed in Web of Science (https://www.webofknowledge.com/) using the query (“phytoplankton” OR “algae” OR “microalgae” OR “picoplankton”) AND (“climate change” OR “global warming” OR “warming” OR “temperature” OR “ocean acidification” OR “CO_2_” OR “carbon dioxide” OR “global change” OR “pCO_2_”) AND (“harmful algal bloom*” OR “harmful alga*” OR “HAB*” OR “toxic” OR “toxin” OR “toxigenic”), yielding a total of 2,927 results on February 22, 2018. This query was set up in such a way that if the authors of the publication classified the studied species as a potential HAB‐forming phytoplankton species, the publication was selected for further reviewing. From these results, first titles and subsequently abstracts were reviewed, which lead to a selection of 144 publications for screening. After careful screening for suitability, a total of 86 publications, containing 169 unique data sets, were included in our database. From these data sets, data on growth rate, cellular toxin content and/or toxicity under different temperature and CO_2_ conditions were extracted, including standard deviations, using Engauge software when needed (Mitchell, Muftakhidinov, & Winchen, 1991). In addition, information on experimental conditions (irradiance, light–dark cycle, salinity, temperature), type of culture (batch, chemostat, etc.) and the site of isolation for each strain was extracted for each experiment as well. Studies with a small sample size (*n* < 2), or unreported sample size, were excluded from the analysis. The database includes only marine and estuarine HAB species, with data acquired through single‐species culture experiments.

### 2.1 Response ratios

For each unique data set, log response ratios of growth rates and/or toxin data were calculated for paired observations of ambient and elevated *p*CO_2_ or temperature. The ambient *p*CO_2_ treatment was categorized as concentrations ranging between 300 and 500 µatm. The elevated *p*CO_2_ treatment corresponded to concentrations more than 1.5 times higher than the ambient treatment, with the highest concentrations at 1,000 µatm, representing a worst‐case future scenario according to the IPCC (2014) (RCP scenario 8.5). On average, *p*CO_2_ ranged from 377 ± 30 to 812 ± 118 µatm, for the ambient and elevated treatments, respectively. As it appeared difficult to define ambient temperature treatments in experimental systems, temperature treatments were defined using two alternative approaches. In the ‘background temperature’ approach, the temperature (±1°C) at which stock cultures were kept (i.e. the background temperature) was used as the ambient treatment. The elevated treatment was subsequently defined as 3–5°C higher than the background temperature, according to RCP scenario 8.5 (IPCC, 2014). In the ‘SST temperature’ approach, the long‐term average spring and summer temperature from the isolation site of each strain was obtained using a high‐resolution SST data set (NOAA long‐term mean 1982–2010; Reynolds et al., 2007). This temperature (±1°C) was then used for the ambient treatment, and the elevated treatment was defined as 3–5°C higher than the ambient. This selection process yielded a total of 26 data sets from 12 publications for CO_2_, and a total of 60 data sets from 24 publications for the background temperature approach and 34 data sets from 14 publications for the SST temperature approach (Table 1). The complete database is deposited in Dryad under https://doi.org/10.5061/dryad.kj67m68.

**Table 1:** Overview of the data sets that were used for the meta‐analysis. All included experiments were performed under nonlimiting nutrient conditions. Salinity is given in PSU and light intensities are given as µmol photons m^−2^ s^−1^

Analysis	Publications	Data sets	Phytoplankton group	Number of species	Salinity range	Light intensity
CO_2_	13	26	22 dinoflagellates	10	10–35	70–250
			2 diatoms			
			1 haptophyte			
			1 raphidophyte			
Temperature background	24	60	48 dinoflagellates	43	10–36	30–350
			4 diatoms			
			4 haptophytes			
			4 raphidophytes			
Temperature SST	14	34	29 dinoflagellates	27	12–36	60–350
			5 haptophytes			

Abbreviations: PSU, practical salinity unit; SST, sea surface temperature.

Log response ratios for growth rate under ambient and high *p*CO_2_ were additionally calculated for non‐HAB phytoplankton species using a database compiled for analysing the effects of climate change variables on phytoplankton in general (see Supplement 1 for further details). The database was filtered to include only data on *p*CO_2_ responses for non‐HAB phytoplankton species under nonlimiting growth conditions. On average, CO_2_ concentrations ranged from 379 ± 28 to 877 ± 245 ppm, for the ambient and elevated treatments respectively. The selection process yielded a total of 24 publications with 53 unique data sets.

Bias‐corrected log response ratios (RR^Δ^) were calculated according to Lajeunesse (2015):

$${\text{RR}}^{\Delta }=\ln\frac{X_{\text{elevated}}}{X_{\text{ambient}}}+\frac{1}{2}\left[\frac{{\left(SD_{\text{elevated}}\right)}^{2}}{n_{\text{elevated}}\times X_{\text{elevated}}}-\frac{{\left(SD_{\text{ambient}}\right)}^{2}}{n_{\text{ambient}}\times X_{\text{ambient}}}\right]$$

$$\begin{array}{c} \text{var}\left({\text{RR}}^{\Delta }\right)=\frac{{\left(SD_{\text{elevated}}\right)}^{2}}{n_{\text{elevated}}\times X_{\text{elevated}}}-\frac{{\left(SD_{\text{ambient}}\right)}^{2}}{n_{\text{ambient}}\times X_{\text{ambient}}}\\ \;\;\;+\frac{1}{2}\left[\frac{{\left(SD_{\text{elevated}}\right)}^{4}}{n_{\text{elevated}}^{2}\times X_{\text{elevated}}^{4}}-\frac{{\left(SD_{\text{ambient}}\right)}^{4}}{n_{\text{ambient}}^{2}\times X_{\text{ambient}}^{4}}\right] \end{array}$$

where *X* represents the mean of the fixed factor of interest (growth rate, toxin content or toxicity), *SD* the standard deviation of that mean and *n* the sample size.

### 2.2 Statistics

Statistical analyses were performed in r version 3.5.0 (R Core Team, 2018). In order to calculate the overall natural log response ratio (RR^Δ^), mixed effect models were fitted to the data set, yielding specific response ratios and their variances using the function *rma.mv* (package ‘metafor’ version 2.0‐0; Viechtbauer, 2010). To correct for dependency of experiments carried out within the same study and/or on organisms from the same genus and/or species, the factors reference, genus and species were modelled as random effects. To attain response ratios per phytoplankton group, separate runs of the model were analysed using phytoplankton group as moderator. To confirm that the variation in experimental *p*CO_2_ and temperature ranges did not influence the strength of the responses, a linear model was fitted between the response ratios and the difference between *p*CO_2_ and temperature in the ambient and elevated treatments. This analysis confirmed that the applied ranges in *p*CO_2_ and temperature did not have an effect on response strength.

To assess the potential role of latitude on species' responses to warming, a linear model was fitted to the RR^Δ^ (for both temperature approaches) and the latitude at which isolations took place. The database was additionally used to determine the optimal growth temperature (*T*_µmax_) for each strain, where only studies were included that measured growth rates at three or more different temperatures. The difference between *T*_µmax_ and the long‐term average spring and summer SST at the isolation site describes the growth potential of HAB species in response to warming.

## 3 RESULTS

**Figure 1:**
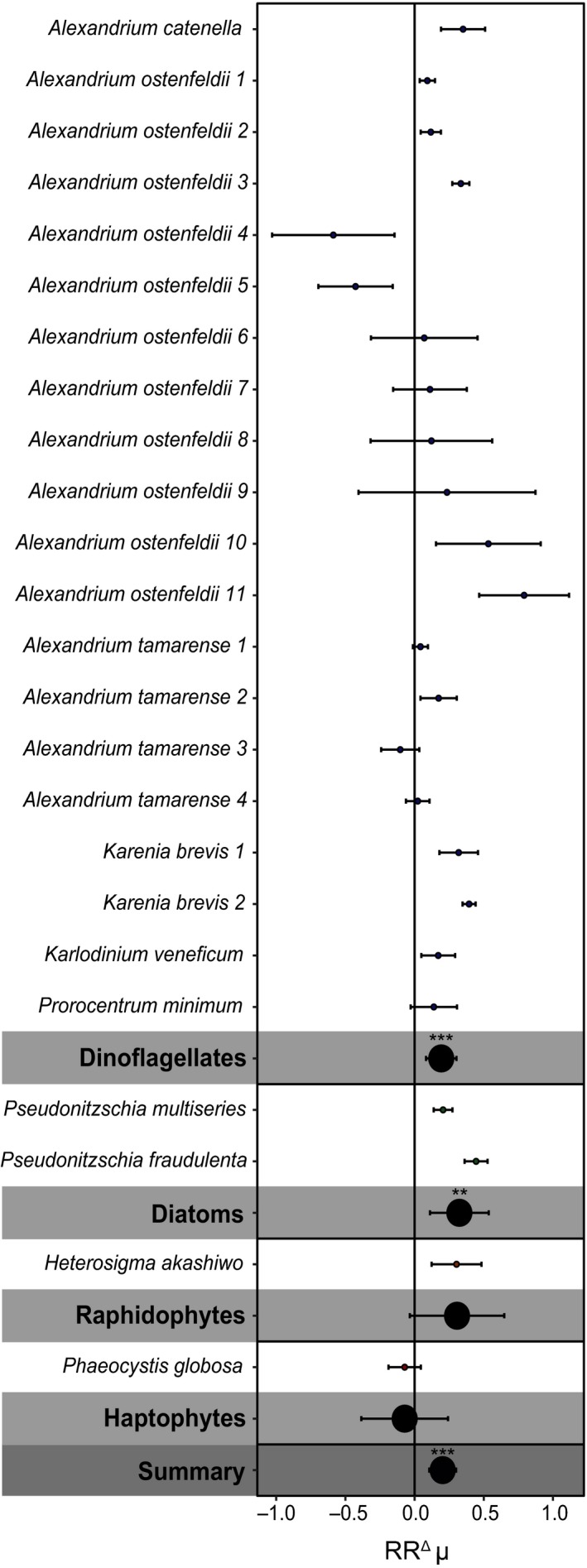
The natural log response ratios (RR^Δ^) for growth rate with elevated *p*CO_2_, shown for individual strains, as well as for the different phytoplankton groups, and a summarized response. Asterisks for the phytoplankton groups and summarized response indicate the level of significance (**p < 0.01, ***p < 0.001) and error bars represent the 95% confidence intervals. See Table S1 for the exact strain identification

**Figure 2:**
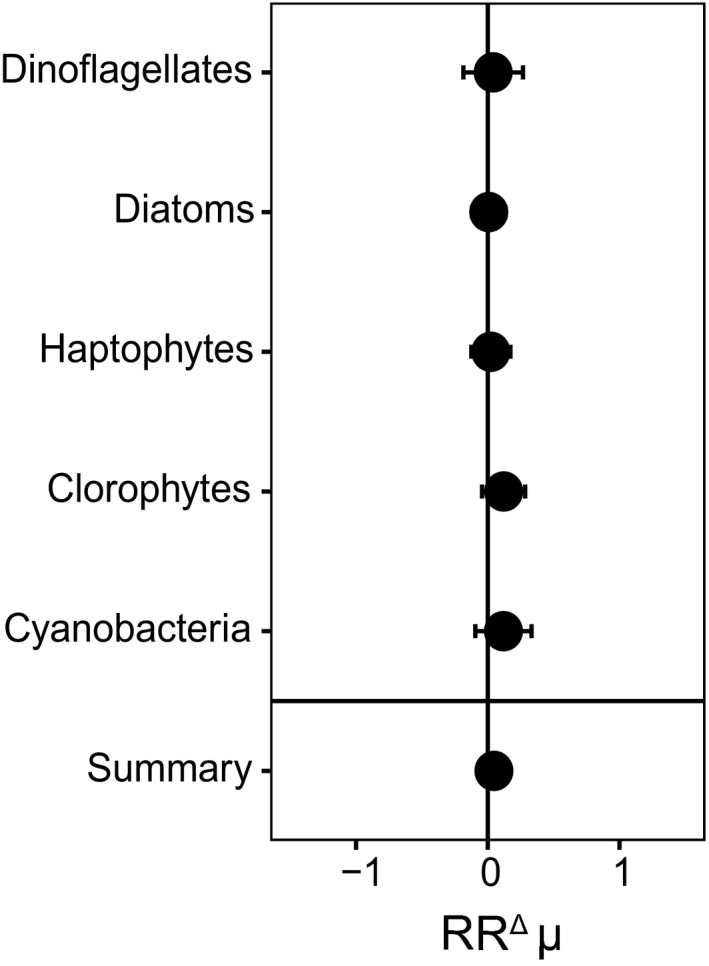
The natural log response ratios (RR^Δ^) for growth rate with elevated *p*CO_2_ for non‐HAB phytoplankton species, shown for the different phytoplankton groups. Error bars represent the 95% confidence intervals

Most of the HAB species that were tested for their response towards elevated *p*CO_2_ and warming were dinoflagellates, and summarized HAB responses were therefore mostly driven by the dinoflagellate responses (Table 1; Figure 1). The effects of elevated *p*CO_2_ on HAB growth rates varied both across and within species, but led to a significant overall increase in growth rate by 20%, which was mainly driven by the similar increase in dinoflagellate growth rates (19%; Figure 1). Moreover, the diatoms, represented by two data sets on *Pseudo‐nitzschia*, also showed a significant increase in growth rate with elevated *p*CO_2_ (by 32%), and the single raphidophyte *Heterosigma akashiwo* tended to increase as well (Figure 1). The non‐HAB phytoplankton species were mostly represented by diatoms and haptophytes, while only two dinoflagellate species were included (Figure 2). Both within and across the different non‐HAB phytoplankton groups no significant response of growth rate to elevated *p*CO_2_ was found (Figure 2).

Overall toxin content did not show a significant response towards higher *p*CO_2_ (*p* > 0.05; Figure S1a). Generally, PSP toxins produced by *Alexandrium* spp. decreased, while karlotoxins produced by *Karlodinium veneficum* and DA produced by *Pseudo‐nitzschia* spp. increased with elevated *p*CO_2_ (Figure S2a). Toxicity responses across dinoflagellate species were also inconsistent and strongly varied (Figure S1b). Specifically, both increases and decreases in toxicity were observed across species and strains (Figure S2b).

**Figure 3:**
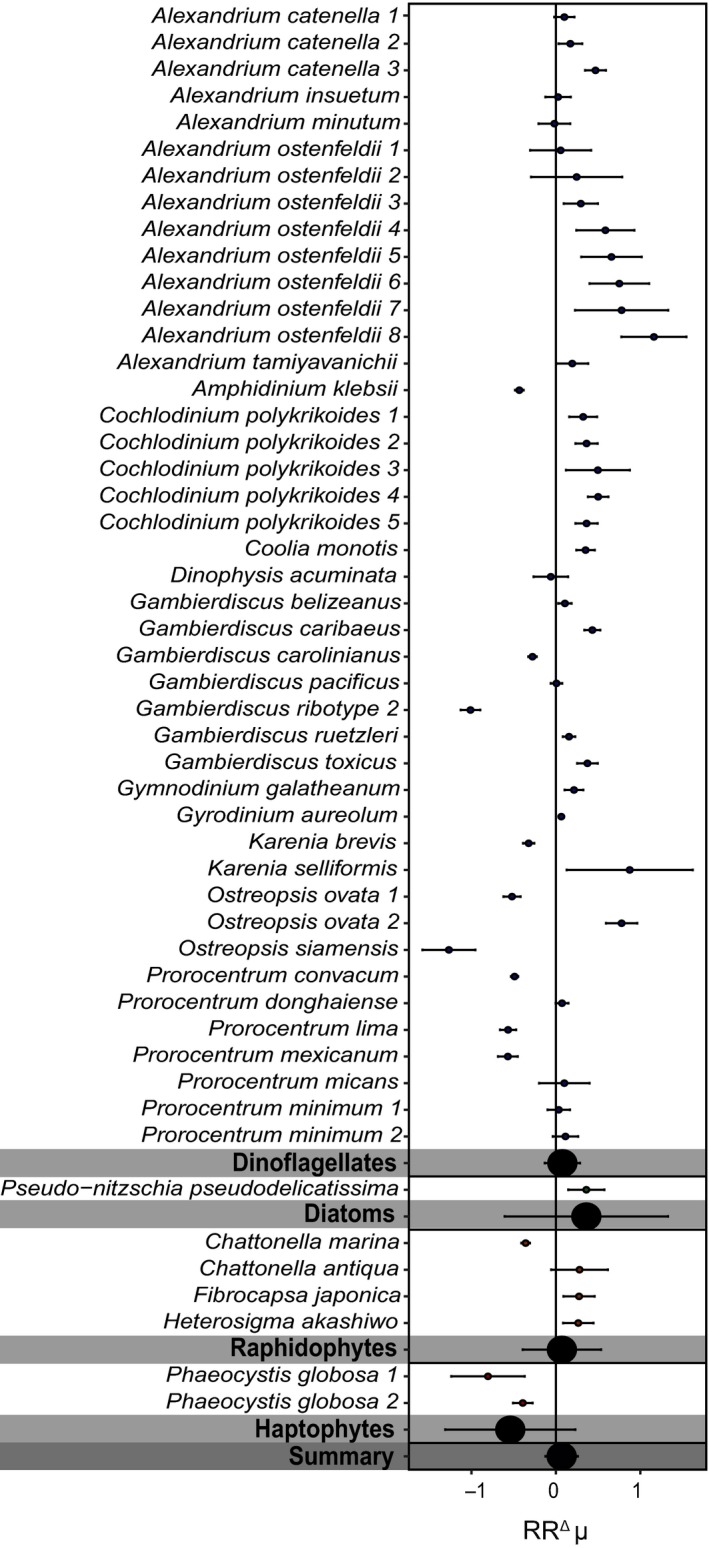
The natural log response ratios (RR^Δ^) for growth rate according to the background temperature approach, shown for individual strains, as well as the different phytoplankton groups, and a summarized response. Error bars represent the 95% confidence intervals. See Table S2 for the exact strain identification

**Figure 4:**
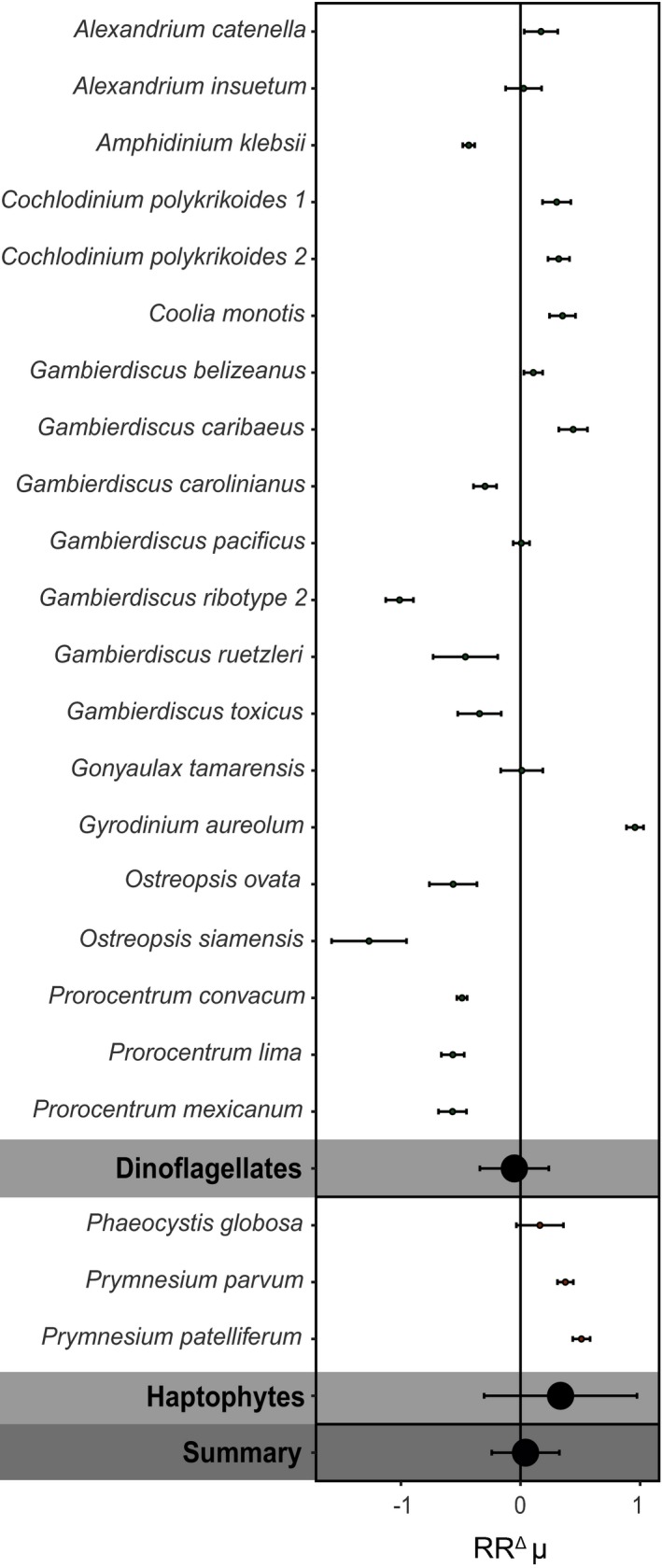
The natural log response ratios (RR^Δ^) for growth rate according to the sea surface temperature approach, shown for individual strains, as well as the different phytoplankton groups, and a summarized response. Error bars represent the 95% confidence intervals. See Table S3 for the exact strain identification

Growth responses towards warming were very variable and remained inconsistent across the different HAB groups, species and strains, independent of the applied temperature approach (*p* > 0.05; Figures 3 and 4). Toxin content and toxicity of dinoflagellate HAB species were also highly variable in the temperature treatments, and did not respond significantly to warming. Toxicity could only be assessed for the SST temperature approach, and tended to decrease with higher temperatures (*p* < 0.05; Figure S1). We note, however, that this was only based on palytoxin and PSP toxin data from two studies (Figure S4). Changes in toxin content could only be assessed based on the background temperature approach (Figure S1). PSP toxin content was highly variable, where *Alexandrium tamiyavanichii*, *Alexandrium minutum* and some *A. ostenfeldii* strains showed an increase with higher temperatures, while *A. tamarense* and other *A. ostenfeldii* strains showed a decrease (Figure S3). Diarrhetic shellfish poisoning toxins produced by *Dinophysis acuminata* and brevetoxins produced by *K. brevis* also showed a slight decrease with temperature, whereas gymnodimines produced by *Karenia selliformis* increased (Figure S3).

**Figure 5:**
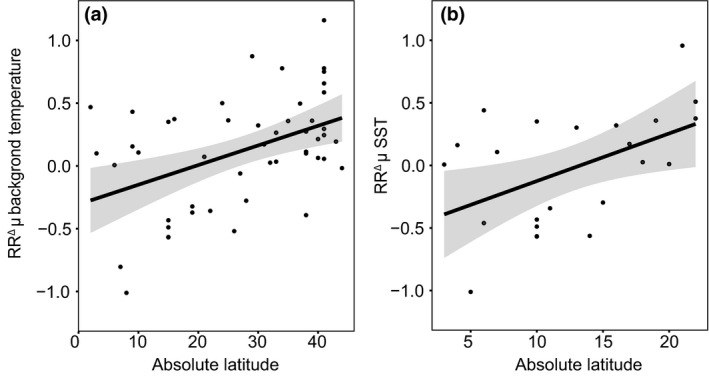
The natural log response ratios (RR^Δ^) for growth rate of individual HAB species and strains plotted against the absolute latitude of the isolation site for (a) the background temperature approach, and (b) the sea surface temperature (SST) approach

**Figure 6:**
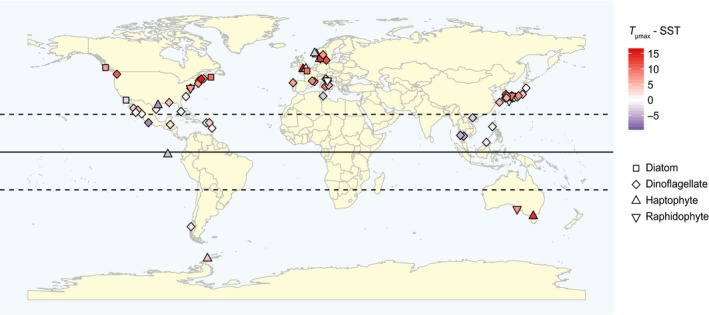
Map presenting the isolation sites of the individual harmful algal bloom species and strains. The differences between the temperature growth optima and the long‐term spring/summer sea surface temperatures (SST) are indicated by the colour gradient. Different phytoplankton groups are indicated with different symbols

Although growth rates did not show a consistent response to warming, we did observe a temperature growth response that depended on latitude. With both the background temperature approach (*R* ^2^ = 0.18, *p* < 0.001) and the SST temperature approach (*R* ^2^ = 0.18, *p* < 0.05), the response of growth rate towards warming increased with latitude (Figure 5). In other words, HAB species at higher latitudes generally responded positively and more strongly to warming as compared to HAB species at lower latitudes that generally did not respond or responded negatively. This is also reflected in the difference between the optimum growth temperature of each strain and the long‐term average spring and summer SST at the isolation site (Figure 6). HAB species originating from higher latitudes grew more often below their temperature optimum than species isolated at lower latitudes. We note that in this analysis HAB species from the tropics, that is, between 23.5° south and 23.5° north of the equator, are under‐represented (see also Figure 6).

## 4 DISCUSSION

Most of the available data on the response of HAB species towards climate change concern dinoflagellates, as many members of this phytoplankton group are responsible for HAB outbreaks. Our analysis revealed a general increase in the growth rate of dinoflagellate HAB species with elevated *p*CO_2_. This suggests that these species are either carbon limited under current atmospheric *p*CO_2_ or possess flexible CCMs (Beardall et al., 2009; Rost et al., 2008). Dinoflagellates exhibit a very inefficient form of RubisCO (type II) with a low specificity towards CO_2_ (Badger et al., 1998; Tortell, 2000), and, therefore, developed highly active CCMs to ensure effective carboxylation (Ratti, Giordano, & Morse, 2007; Rost, Richter, Riebesell, & Hansen, 2006). More active CCMs, including those of dinoflagellates, were also shown to be downregulated with elevated *p*CO_2_ (Eberlein et al., 2016; Raven, Giordano, Beardall, & Maberly, 2011; Rost et al., 2008; Van de Waal et al., 2019). Earlier findings suggested that elevated *p*CO_2 _may lead to reallocation of energy and/or resources from the operation of CCMs to nitrogen acquisition in two dinoflagellates, resulting in increased quota of nitrogen‐rich functional compounds such as PSP toxins and chlorophyll‐a (Eberlein et al., 2016). Thus, the observed CO_2_‐driven increases in growth rate of dinoflagellates may possibly have resulted from downregulation of their CCMs, where energy and/or resources were reallocated to growth‐related processes.

Besides dinoflagellates, some other HAB species with distinct carbon acquisition characteristics were included in our analysis as well. For instance, the raphidophyte *H. akashiwo* was shown to depend on diffusive CO_2_ uptake alone, and its seemingly higher growth rate under elevated *p*CO_2_ is thus likely caused by direct fuelling of photosynthesis with more CO_2_ (Fu et al., 2008; Nimer, Iglesias‐Rodriguez, & Merrett, 1997). Diatoms generally possess CCMs with lower activity and plasticity (Badger et al., 1998; Tortell, 2000; Van de Waal et al., 2019). However, both *Pseudo‐nitzschia* species increased their growth rate under higher *p*CO_2_. This suggests that despite lower CCM activity and plasticity, *Pseudo‐nitzschia* is able to regulate its carbon acquisition and increase its growth with elevated *p*CO_2_ (Burkhardt, Amoroso, Riebesell, & Sültemeyer, 2001; Trimborn et al., 2008). The haptophyte *Phaeocystis globosa* showed no significant change in growth rate with more CO_2_. This may be explained by its possession of effective CCMs saturating photosynthetic carbon fixation at present‐day CO_2_ levels. Moreover, *P. globosa* appears unable to adjust its CCMs, as carbon uptake affinities did not change as a function of CO_2_ (Rost, Riebesell, Burkhardt, & Sültemeyer, 2003). We note, however, that for HAB species other than dinoflagellates, the number of studies is limited, and future studies on more species from these phytoplankton groups are required to better assess their responses as well as the overall responses of HAB species to elevated *p*CO_2_.

The observed increases in HAB growth rates in response to elevated *p*CO_2_ may potentially warn for more HAB events in future coastal waters. However, whether HAB species will become more prominent not only depends on their intrinsic growth rates, but also on their interactions within the food web. Loss rates, either through grazing or by parasite infections, and alterations in these loss rates through climate change, will impact future HAB development as well (Salomon & Umai, 2006; Schultz & Kiørboe, 2009; Wells et al., 2015). Zooplankton grazing rates may, for instance, increase as a result of warming (O'Connor, Piehler, Leech, Anton, & Bruno, 2009; Sommer & Lengfellner, 2008). This may lead to a reduction in HAB frequency, magnitude and/or duration, as a direct consequence of increased grazing rates, but can also lead to an increase in HAB development, if selective grazing more strongly affects competing non‐HAB phytoplankton species.

Climate change may also alter growth rates of competing phytoplankton species, which in turn may impact potential HAB development. Here, we also assessed the effects of elevated *p*CO_2_ on non‐HAB phytoplankton species, where no significant change in growth rate was observed (*p* > 0.05; Figure 2). Most of the non‐HAB species included in this analysis were diatoms or haptophytes, and both these phytoplankton groups were shown to possess CCMs with lower activity and plasticity (Raven et al., 2011; Reinfelder, 2011; Van de Waal et al., 2019). This difference in representation of phytoplankton groups in the HAB and non‐HAB CO_2_ analyses may explain the differences in their general growth responses. However, the two harmful diatom species did increase their growth rates, while the two nonharmful dinoflagellate species did not respond to more *p*CO_2_. Although these sample sizes are too small to draw conclusions, they do raise the question whether toxin‐producing phytoplankton species may somehow benefit more from elevated *p*CO_2_ than their non‐toxic counterparts. Higher growth rates in response to CO_2_ may nonetheless present a competitive advantage for dinoflagellates in a future ocean, which may also lead to more dinoflagellate‐related HAB events.

There was no significant overall response in growth rate to higher temperatures for both temperature approaches (Figures 3 and 4). We did, however, observe a significant correlation between latitude and RR^Δ^ (Figure 5). More specifically, HAB species from higher latitudes responded mostly positive to higher temperatures, while isolates from lower latitudes responded more negatively (Figure 5). These findings are in line with earlier work across marine phytoplankton species, showing that species from the tropics grow at their temperature optimum, while species at higher latitudes grow below their temperature optimum (Thomas et al., 2012). Consequently, phytoplankton species at higher latitudes might benefit from warming, assuming that their growth is not limited by other environmental factors. To assess the role of latitude on the potential of HAB species to increase growth with warming, we compared their optimum growth temperature to the average spring/summer SSTs at the site of isolation (Figure 6). The growth optimum lies well above the average SST at higher latitudes, while this is mostly not the case for lower latitudes. HAB species, therefore, have a greater potential to proliferate with warming at higher latitudes, where temperature increases will also be most pronounced (IPCC, 2014).

The temperature response of HAB species seems to be comparable with the response of other phytoplankton species (Thomas et al., 2012). It is, therefore, unlikely that growth of HAB species will be selectively favoured by warming as compared to non‐toxic phytoplankton species. Modelling studies, however, have projected more intense and longer lasting HABs in response to warming, particularly at higher latitudes (Glibert et al., 2014; Gobler et al., 2017; Moore et al., 2015). Moreover, a modelling study from the Caribbean predicts that shifts in the phytoplankton community composition may stabilize or slightly lower the risks of ciguatera fish poisoning in the Caribbean Sea, but increases its risk in more northern regions (Kibler et al., 2015). Tropical HAB species may thus respond negatively to warming at their current locations, yet can impose risks at higher latitudes. Changes in the proliferation of HABs will not only depend on growth responses of HAB species within existing communities, but also on shifts in phytoplankton community composition, which will change as the distribution of ocean temperatures changes (Anderson et al., 2012; Hallegraeff, 2010; Heisler et al., 2008). Indeed, various studies have highlighted potential range shifts of HAB species, generally from tropical to temperate regions (Kibler, Litaker, Holland, Vandersea, & Tester, 2012; Kibler et al., 2015; Tester, Feldman, Nau, Kibler, & Wayne Litaker, 2010). Furthermore, not all ocean regions may experience warming as a result of climate change (Bakun, 1990). Along the South American Pacific gradient, for instance, considerable cooling occurred over the last few decades (Baumann & Doherty, 2013). This may also alter phytoplankton community composition, with putative shifts in the proliferation of HABs.

Responses of toxin production and toxicity to higher temperatures and elevated *p*CO_2_ were highly variable (Figure S1). This is possibly due to the variety of toxins included in the analysis, but can also be attributed to the limited availability of data in this field. Our data collection particularly revealed a knowledge gap regarding the effects of warming on toxin content and toxicity. Toxin content and toxicity showed increases and decreases with warming and elevated *p*CO_2_, but very little is known about the metabolic pathways underlying these changes. The observed decrease in PSP content under elevated *p*CO_2_ may be related to a downregulation of amino acid transport and metabolism (Van de Waal et al., 2014), as arginine is an important precursor for PSP toxins (Shimizu, 1996). This may, however, not always be the case as some *Alexandrium* species showed an increase in toxicity with more CO_2_ (Kremp et al., 2012; Pang et al., 2017; Tatters et al., 2013). Higher toxin content as a function of CO_2_, as observed for karlotoxin and DA, may be a consequence of excess carbon supply that is shunted towards synthesis of these carbon‐rich compounds (Fu, Place, Garcia, & Hutchins, 2010; Fu et al., 2012; Sun et al., 2011).

Toxin content, and particularly that of PSP toxin, was shown to be inversely related to growth rate, where slower growing cells tend to be larger and contain more toxins (Cembella, 1998; Proctor, Chan, & Trevor, 1975; but see Van de Waal et al., 2013). We did not find a significant correlation between the RR^Δ^ of *Alexandrium* growth rates and PSP toxin contents when changes were a function of temperature (*R* ^2^ = 0.10, *p* = 0.19). However, it was significant when the changes were a function of CO_2_ (*R* ^2^ = 0.20, *p* = 0.046). Thus, CO_2_‐driven increases in *Alexandrium* growth rates may be accompanied by increases in their toxin content as well, potentially resulting from CO_2_‐dependent increases in nitrogen acquisition (Eberlein et al., 2016). Although warming‐induced changes in growth rates were not accompanied by consistent shifts in toxin content, temperature can alter toxin content directly through changes in toxin biosynthesis. For example, lower temperatures caused a decrease in PSP toxin content by suppressing toxin synthesis or a related process (Lim et al., 2006). In contrast, lower temperatures may cause an increased PSP toxin content by reducing protein synthesis, which may leave a surplus of arginine within the cell that can be used for toxin synthesis (Anderson, Kulis, Sullivan, Hall, & Lee, 1990; Usup, Kulis, & Anderson, 1994). Predictions on how HAB toxicity may change in response to climate change remains a challenge, as responses not only vary between different types of toxins, but responses within toxin synthesis pathways vary between different species and strains as well.

Climate change will lead to elevated *p*CO_2_ concomitantly with higher temperatures. Here, we tested the effects of both factors separately, since only few studies performed interaction experiments with HAB species. In experiments testing these combined factors, growth rates were generally higher as compared to either the warming or the elevated *p*CO_2_ treatments, but were not additive (Errera, Yvon‐Lewis, Kessler, & Campbell, 2014; Fu et al., 2008; Kremp et al., 2012; Tatters et al., 2013). For example, an increase in elevated *p*CO_2_ was further stimulated with warming, while the effect of warming alone was stronger than this interactive effect (Kremp et al., 2012). Thus, the interactive effect of both climate change factors is generally in line with the effects of warming or elevated *p*CO_2_ alone, although the strength of the responses may vary.

Although controlled single strain experiments strongly support our understanding on the response of a species towards a climate change factor, how HABs will ultimately respond to climate change cannot be directly extrapolated. Though HABs are mostly dominated by one phytoplankton species, culture experiments are not fully able to reflect the vast complexity of natural communities, as discussed above, nor that of populations. Single‐strain culture experiments, for instance, do not account for intraspecific genetic and phenotypic variation in HAB species. Indeed, various harmful algal populations were shown to possess a very high genetic and phenotypic variation (Alpermann, Tillmann, Beszteri, Cembella, & John, 2010; Brandenburg et al., 2018; Medlin, Lange, & Nothig, 2000), which will allow them to adapt to changing environmental conditions (Violle et al., 2012). This potential for rapid evolution may even occur on ecological timescales (Carroll, Hendry, Reznick, & Fox, 2007), and it remains to be tested whether this could lead to faster and/or stronger increases in growth of HAB species with elevated *p*CO_2_ and warming.

The empirical data presented here supports earlier findings that HABs, in particular caused by dinoflagellates, can potentially become more prominent as a consequence of climate change (Glibert et al., 2014; Gobler et al., 2017; Kibler et al., 2015; Moore et al., 2015). However, the development of HABs is not determined by climatic conditions alone. Bloom development depends strongly on nutrient availability, and eutrophication was shown to be an important driver for the observed increase in outbreaks of some HAB species in coastal waters over the last decades (Anderson et al., 2002; Brandenburg et al., 2017; Glibert & Burkholder, 2006; Glibert et al., 2005; Parsons & Dortch, 2002; Smayda, 1997; Trainer et al., 2003). We furthermore showed that while temperature responses are comparable across phytoplankton species, elevated *p*CO_2_ promoted the growth of dinoflagellate HAB species more strongly than other phytoplankton species. Thus, our findings highlight the potential for an increase in dinoflagellate HAB development, particularly in temperate regions.
