# Supplementary material for: Meta‐analysis reveals enhanced growth of marine harmful algae from temperate regions with warming and elevated CO2 levels
Source: Glob Chang Biol. 2019 Jun 17;25(8):2607–18. doi: 10.1111/gcb.14678 (PMC6851565; doi:10.1111/gcb.14678)
Supplement: Supplementary file 1 [file GCB-25-2607-s001.docx]

**Supplementary Figures**

**
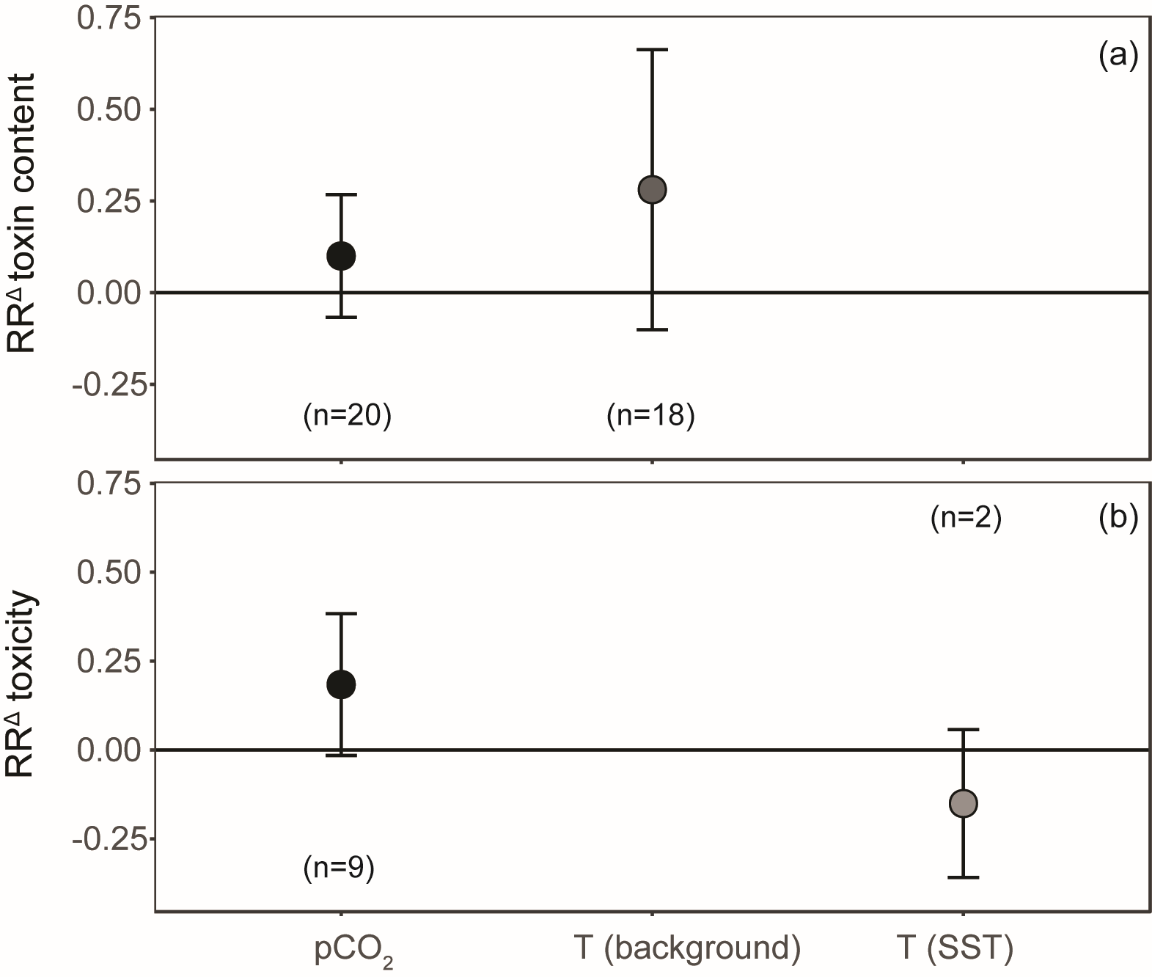
**

**Figure S1**. The natural log response ratios (RR^Δ^) for (a) toxin content, and (b) toxicity with elevated *p*CO_2_ and temperature, following the background temperature approach and the SST temperature approach. Error bars represent the 95% confidence intervals.


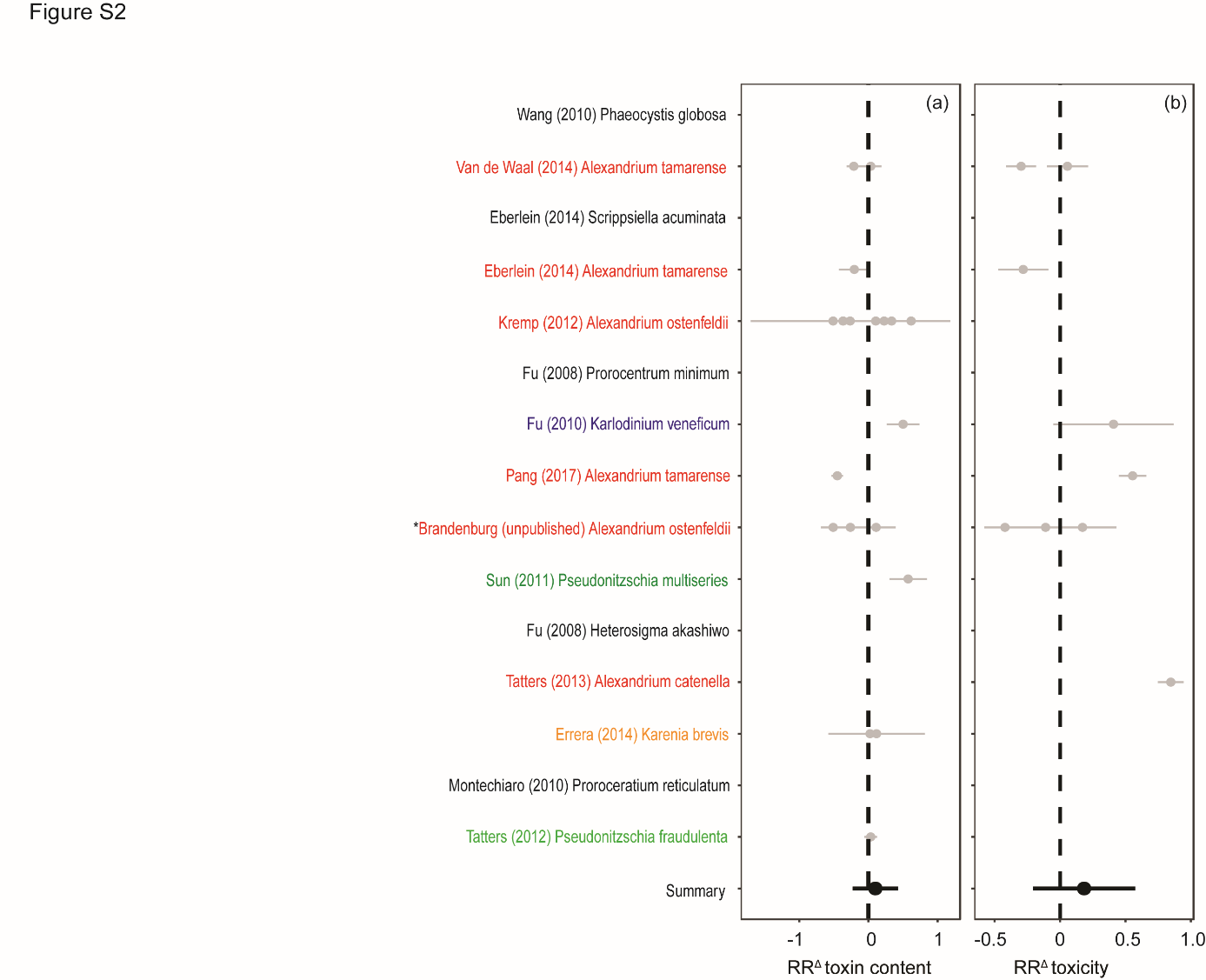


**Figure S2**. The natural log response ratios (RR^Δ^) for (a) toxin content, and (b) toxicity with elevated *p*CO_2_ for the individual HAB species and strains. Red indicates PSP producers, green DA producers, orange NSP producers, and dark blue karlotoxin producers. *details on the unpublished data can be found in supplement 2.


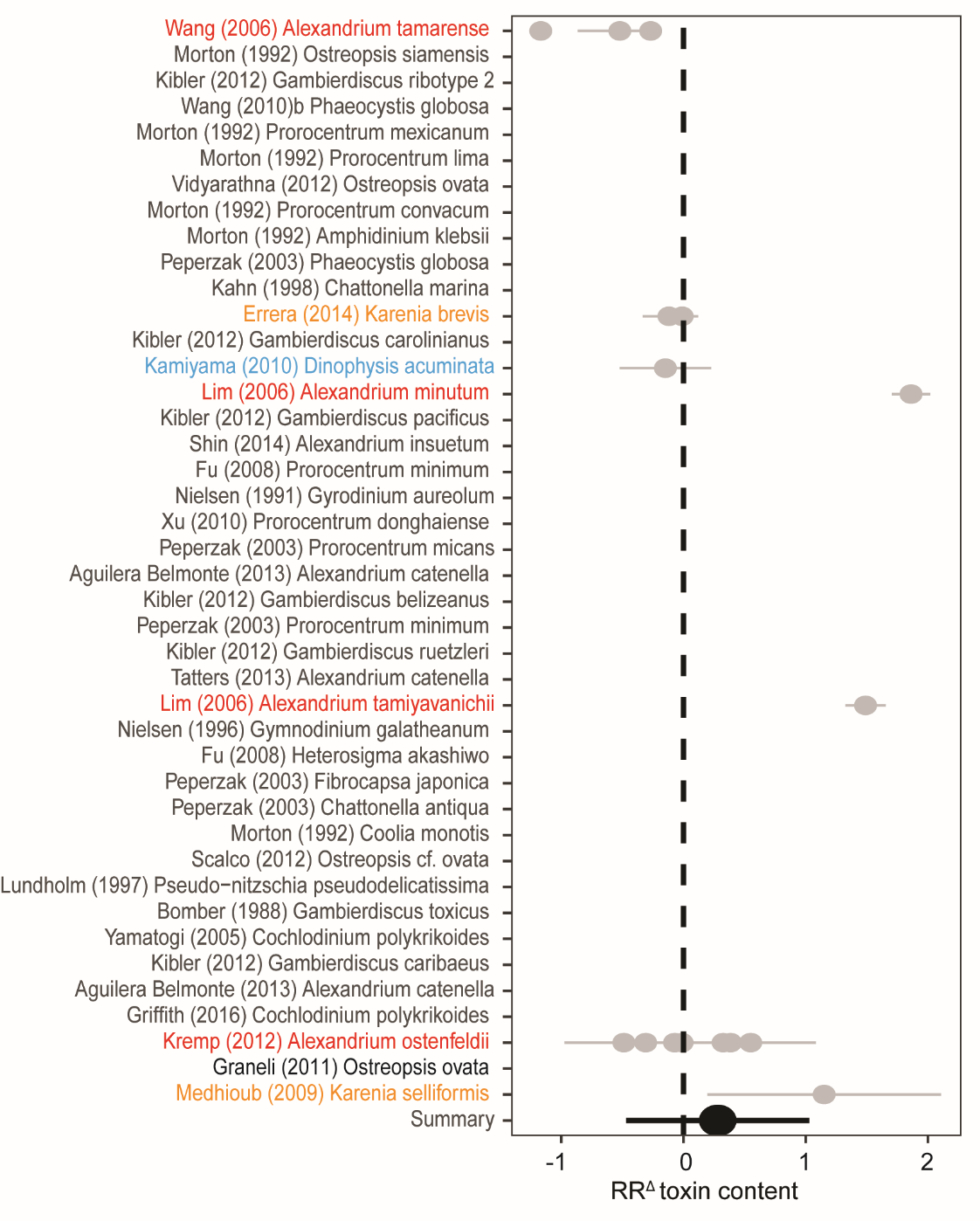


**Figure S3**. The natural log response ratios (RR^Δ^) for toxin content using the background temperature approach for the individual HAB species and strains. Red indicates PSP producers, and purple palytoxin producers.


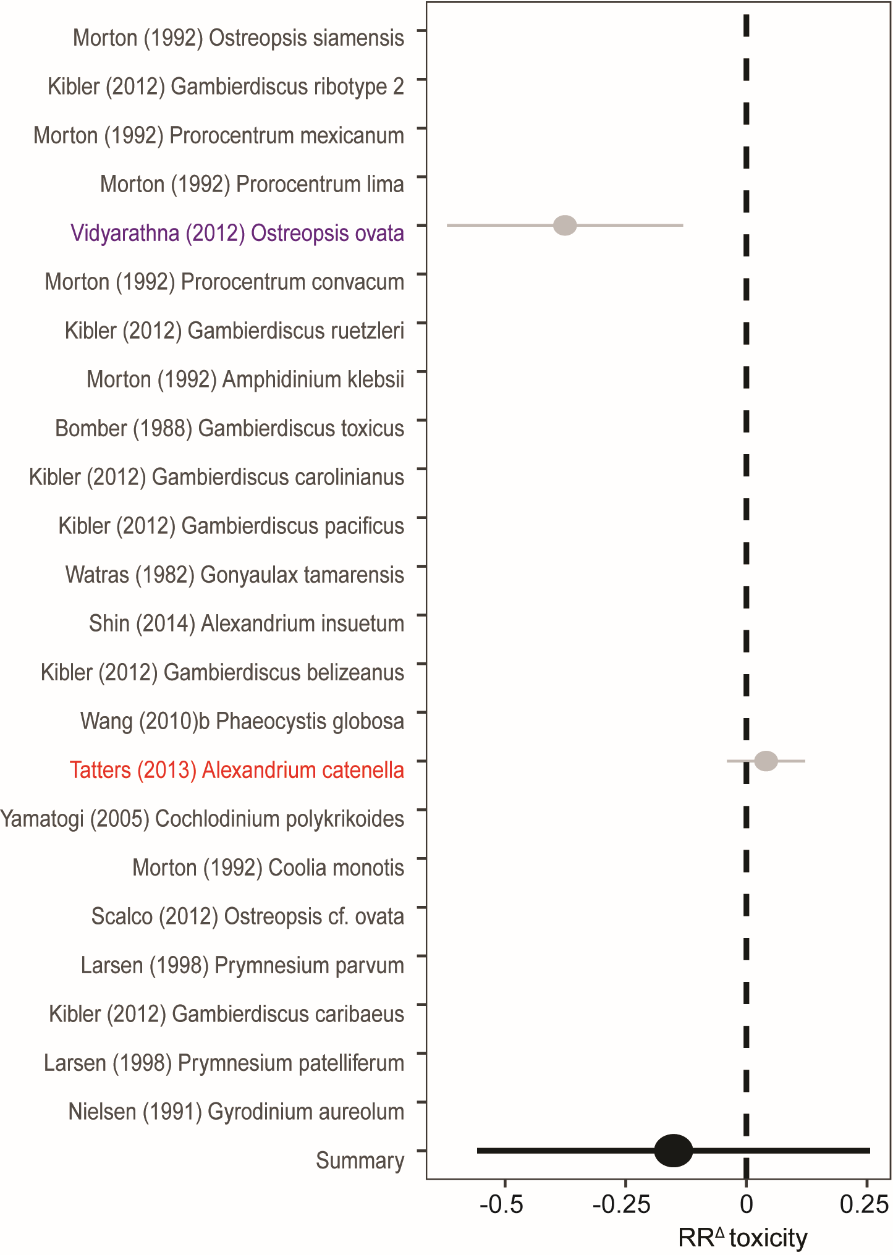


**Figure S4**. The natural log response ratios (RR^Δ^) for toxicity using the SST temperature approach for the individual HAB species and strains. Red indicates PSP producers, orange NSP producers, and light blue DSP producers.

**Supplement 1: non-HAB phytoplankton species**

For the growth data of non-HAB species in response to elevated *p*CO_2_ a systematic literature review was carried out on the Web of Science (<https://www.webofknowledge.com/>) on 19-02-2016 using the query (stoichiometry OR “nutrient stoichiometry” OR “CNP stoichiometry” OR “chemical composition” OR “nutritional quality” OR “nutrient composition” OR “elemental composition” OR C:N:P OR carbon:nitrogen:phosphorus) AND (phytoplankton OR algae OR microalgae OR algal OR picoplankton) AND (“climate change” OR “ocean acidification” OR CO2 OR “carbon dioxide” OR “global change” OR pCO2). The resulting publications were screened on titles, abstracts, graphs and tables. Freshwater species, macroalgal species and data from community studies were excluded from analysis, as well as studies carried out under nutrient limited conditions (as described in the original paper) and studies where sample size was small (n<2), or not reported. From the remaining publications, data was extracted with use of Plotdigitizer (Huwaldt 2013) and Engauge (Mitchell *et al.* 2017). Standard deviations were either extracted from the papers or calculated from the data and sample size. Species names were checked with Algaebase (Guiry and Guiry 2017) and updated to their latest taxonomy if necessary. This process rendered 24 publications with 53 independent studies that contained information on CO_2_ effects on growth rates and elemental composition of phytoplankton.

**Literature**

Guiry, M. D., and G. M. Guiry. 2017. AlgaeBase. World-wide electronic publication, National University of Ireland, Galway, <http://www.algaebase.org>.

Huwaldt, J. A. 2013. Plotdigitizer Version 2.6.3. <http://plotdigitizer.sourceforge.net/>.

Mitchell, M., B. Muftakhidinov, and T. Winchen. 1991. Engauge Digitizer Software Version 2. Webpage: <http://markummitchell.github.io/engauge-digitizer>, Last Accessed: May 9, 2017.

**Supplement 2: details on unpublished data**

*Culturing*

Three datasets on *Alexandrium ostenfeldii* strains (AON13, AON15, AON5.26) used in this analysis were not published yet. The three strains were isolated from the Ouwerkerkse Kreek, the Netherlands (51˚62’N, 3˚99’E), a brackish water creek, during summer blooms in 2015 (AON5.26) and 2016 (AON13; AON15). The strains were cultured at the Netherlands Institute of Ecology in ½K – medium (after Keller et al. 1987), using 0.2 µm sterilized North Sea water adjusted to a salinity of 10 by dilution with demineralized water. Culture medium was pre-aerated with air containing *p*CO_2_ of 400 µatm (ambient CO_2_ treatment) and 1000 µatm (high CO_2_ treatment). These concentrations were obtained by mixing pressurized air with CO_2_ (100%) using mass flow controllers (SLA5800 series, Brooks Instruments, Hatfield, US). CO_2_ concentrations were verified by a nondispersive infrared analyzer system (LI-820, LI-COR Biosciences, Bad Homburg, Germany). Before the experiment, cultures were first acclimated to the experimental CO_2_ concentrations for 14 days, representing at least 3 generations. Cultures were subsequently grown in 2 L round bottom flasks in triplicates, at a temperature of 18˚C and an incident light intensity of 85 µmol photons m^-2^ s^-1^, with a light dark cycle of 16:8 h.

*Growth rate*

Cell densities were estimated by taking a sample for cell counts every two or three days. A sample of 5 mL was taken and fixed with Lugol’s iodine solution (Lugol) to a final concentration of 1% and stored in the dark at 4 ˚C until analysis. *A. ostenfeldii* cells were counted on an inverted microscope (DMI 4000B; Leica Microsystems CMS GmbH, Mannheim, Germany). Specific growth rates (µ) were calculated for each replicate of each strain by fitting an exponential function through all cell counts over time, following:

$N_{t}= N_{0}\exp^{\mu t}$

where N_t_ refers to the cell concentrations at time t, and N_0_ to the cell concentrations at the start of each experiment.

**Table S1**. Overview of the corresponding study and strain names for the species used in the *p*CO_2_ analysis (Fig. 1).

| Species | Reference | Strain |
| --- | --- | --- |
| Alexandrium catenella | Tatters (2013) | A-11c |
| Alexandrium ostenfeldii 1 | Brandenburg (unpublished) | AON13 |
| Alexandrium ostenfeldii 2 | Brandenburg (unpublished) | AON15 |
| Alexandrium ostenfeldii 3 | Brandenburg (unpublished) | AON5.26 |
| Alexandrium ostenfeldii 4 | Kremp (2012) | AO07 |
| Alexandrium ostenfeldii 5 | Kremp (2012) | AO05 |
| Alexandrium ostenfeldii 6 | Kremp (2012) | AO08 |
| Alexandrium ostenfeldii 7 | Kremp (2012) | AO06 |
| Alexandrium ostenfeldii 8 | Kremp (2012) | AO01 |
| Alexandrium ostenfeldii 9 | Kremp (2012) | AO03 |
| Alexandrium ostenfeldii 10 | Kremp (2012) | AO02 |
| Alexandrium ostenfeldii 11 | Kremp (2012) | AO04 |
| Alexandrium tamarense 1 | Eberlein (2014) | Alex5 |
| Alexandrium tamarense 2 | Pang (2017) | ATDH |
| Alexandrium tamarense 3 | Van de Waal (2014) | Alex2 |
| Alexandrium tamarense 4 | Van de Waal (2014) | Alex5 |
| Karenia brevis 1 | Errera (2014) | SP1 |
| Karenia brevis 2 | Errera (2014) | CCFWC268 |
| Karlodinium veneficum | Fu (2010) | CCMP2936 |
| Prorocentrum minimum | Fu (2008) | CCMP2233 |
| Pseudonitzschia multiseries | Sun (2011) | 2708 |
| Pseudonitzschia fraudulenta | Tatters (2012) | WWA7 |
| Heterosigma akashiwo | Fu (2008) | CCMP2393 |
| Phaeocystis globosa | Wang (2010) | CCMP1528 |

**Table S2**. Overview of the corresponding study and strain names for the species used in the background temperature analysis (Fig. 3a).

| Species | Reference | Strain |
| --- | --- | --- |
| Alexandrium catenella | Aguilera Belmonte (2013) | PFB37 |
| Alexandrium catenella | Tatters (2013) | A-11c |
| Alexandrium catenella | Aguilera Belmonte (2013) | PFB41 |
| Alexandrium insuetum | Shin (2014) |  |
| Alexandrium minutum | Lim (2006) | AmKB02 |
| Alexandrium ostenfeldii | Kremp (2012) | AO05 |
| Alexandrium ostenfeldii | Kremp (2012) | AO03 |
| Alexandrium ostenfeldii | Kremp (2012) | AO02 |
| Alexandrium ostenfeldii | Kremp (2012) | AO07 |
| Alexandrium ostenfeldii | Kremp (2012) | AO04 |
| Alexandrium ostenfeldii | Kremp (2012) | AO06 |
| Alexandrium ostenfeldii | Kremp (2012) | AO01 |
| Alexandrium ostenfeldii | Kremp (2012) | AO08 |
| Alexandrium tamiyavanichii | Lim (2006) | AcMS01 |
| Amphidinium klebsii | Morton (1992) | AKI00A |
| Cochlodinium polykrikoides | Yamatogi (2005) | 03H1 |
| Cochlodinium polykrikoides | Yamatogi (2005) | KG8-ND14 |
| Cochlodinium polykrikoides | Griffith (2016) | CP-1 |
| Cochlodinium polykrikoides | Yamatogi (2005) | 02B2 |
| Cochlodinium polykrikoides | Yamatogi (2005) | 02B1 |
| Coolia monotis | Morton (1992) | CM300A |
| Dinophysis acuminata | Kamiyama (2010) |  |
| Gambierdiscus belizeanus | Kibler (2012) | CCMP399 |
| Gambierdiscus caribaeus | Kibler (2012) | NOAA19 |
| Gambierdiscus carolinianus | Kibler (2012) | NOAA6 |
| Gambierdiscus pacificus | Kibler (2012) | CCMP1650 |
| Gambierdiscus ribotype 2 | Kibler (2012) | CCMP1655 |
| Gambierdiscus ruetzleri | Kibler (2012) | NOAA8 |
| Gambierdiscus toxicus | Bomber (1988) | GT600 |
| Gymnodinium galatheanum | Nielsen (1996) | KT76E |
| Gyrodinium aureolum | Nielsen (1991) | Hulburt |
| Karenia brevis | Errera (2014) | CCFWC268 |
| Karenia selliformis | Medhioub (2009) | GM94GAB |
| Ostreopsis ovata | Vidyarathna (2012) | S0662 |
| Ostreopsis ovata | Graneli (2011) | KAC85 |
| Ostreopsis siamensis | Morton (1992) | OS IOOA |
| Prorocentrum convacum | Morton (1992) | PC100A |
| Prorocentrum donghaiense | Xu (2010) |  |
| Prorocentrum lima | Morton (1992) | PL100A |
| Prorocentrum mexicanum | Morton (1992) | PM200A |
| Prorocentrum micans | Peperzak (2003) | CCMP1589 |
| Prorocentrum minimum | Fu (2008) | CCMP2233 |
| Prorocentrum minimum | Peperzak (2003) | BAHME66 |
| Pseudo-nitzschia pseudodelicatissima | Lundholm (1997) | LØgst J |
| Chattonella marina | Kahn (1998) |  |
| Heterosigma akashiwo | Fu (2008) | CCMP2393 |
| Chattonella antiqua | Peperzak (2003) | CaKA84 |
| Fibrocapsa japonica | Peperzak (2003) | FjW420 |
| Phaeocystis globosa | Wang (2010)b | CCMP1528 |
| Phaeocystis globosa | Peperzak (2003) | Ph91 |

**Table S3**. Overview of the corresponding study and strain names for the species used in the SST temperature analysis (Fig. 3b).

| Species | Study | Strain |
| --- | --- | --- |
| Alexandrium catenella | Tatters (2013) | A-11c |
| Alexandrium insuetum | Shin (2014) |  |
| Amphidinium klebsii | Morton (1992) | AKI00A |
| Cochlodinium polykrikoides | Yamatogi (2005) | KG8-ND14 |
| Cochlodinium polykrikoides | Yamatogi (2005) | 03H1 |
| Coolia monotis | Morton (1992) | CM300A |
| Gambierdiscus belizeanus | Kibler (2012) | CCMP399 |
| Gambierdiscus caribaeus | Kibler (2012) | NOAA19 |
| Gambierdiscus carolinianus | Kibler (2012) | NOAA6 |
| Gambierdiscus pacificus | Kibler (2012) | CCMP1650 |
| Gambierdiscus ribotype 2 | Kibler (2012) | CCMP1655 |
| Gambierdiscus ruetzleri | Kibler (2012) | NOAA8 |
| Gambierdiscus toxicus | Bomber (1988) | GT600 |
| Gonyaulax tamarensis | Watras (1982) | GTMP |
| Gyrodinium aureolum | Nielsen (1991) | Hulburt |
| Ostreopsis ovata | Vidyarathna (2012) | S0662 |
| Ostreopsis siamensis | Morton (1992) | OS IOOA |
| Prorocentrum convacum | Morton (1992) | PC100A |
| Prorocentrum lima | Morton (1992) | PL100A |
| Prorocentrum mexicanum | Morton (1992) | M200A |
| Phaeocystis globosa | Wang (2010)b | CCMP1528 |
| Prymnesium parvum | Larsen (1998) | RL10parv93 |
| Prymnesium patelliferum | Larsen (1998) | RHpat93 |
